# Berry Anthocyanins and Their Metabolites in Endothelial Dysfunction Associated with Diabetes and Cardiometabolic Risk

**DOI:** 10.3390/molecules31203618

**Published:** 2026-10-11

**Authors:** Małgorzata Elżbieta Zujko

**Affiliations:** Department of Food Biotechnology, Faculty of Health Sciences, Medical University of Białystok, Szpitalna 37, 15-295 Białystok, Poland; malgorzata.zujko@umb.edu.pl

**Keywords:** anthocyanins, berries, diabetes mellitus, endothelial dysfunction, phenolic metabolites

## Abstract

Diabetes-related endothelial dysfunction involves oxidative stress, impaired nitric oxide (NO) signaling, and inflammation. Because ingested berry anthocyanins undergo extensive metabolism, circulating conjugates and phenolic products may contribute to vascular responses. This critical narrative review searched MEDLINE/PubMed, Web of Science, and Scopus through 1 July 2026 and examined citation trails. Among 298 database records, 253 were unique. The screening log selected 29 reports for full-text assessment; 26 were included and three excluded after full-text analysis. Four reports entered through citation tracking. The 30 included reports comprised eight cell-based, twelve animal or mixed, and ten human reports; two human reports analyzed related participants. Experimental studies associated berry preparations or metabolites with lower measured reactive oxygen species, NADPH oxidase (NOX) expression, and inflammatory markers, sometimes alongside preserved NO signaling or vascular relaxation. Expression changes do not establish NOX inhibition or nuclear factor kappa B (NF-κB)-dependent mediation. Several small phenolic products lack anthocyanin-specific provenance. Human vascular findings in cardiometabolic risk populations were mixed, and no included berry intervention in established diabetes used a direct endothelial endpoint. Current evidence supports mechanistic plausibility but does not establish clinical efficacy in diabetes or attribute whole-berry effects to anthocyanins alone.

## 1. Introduction

Diabetes mellitus is a major cause of microvascular complications, including retinopathy and nephropathy, and is an important risk factor for macrovascular diseases such as peripheral arterial disease and atherosclerotic cardiovascular disease. Endothelial dysfunction is an early feature of these complications and is characterized by impaired endothelium-dependent vasodilation, reduced nitric oxide (NO) bioavailability, and a pro-inflammatory, pro-oxidant, and pro-thrombotic endothelial phenotype. Chronic hyperglycemia, insulin resistance, dyslipidemia, and lipotoxicity interact to promote reactive oxygen species (ROS) production, signaling through the receptor for advanced glycation end products (RAGE), protein kinase C (PKC) activation, mitochondrial dysfunction, and vascular inflammation [1,2,3].

NADPH oxidase (NOX) enzymes are important sources of vascular ROS in diabetes. Sustained or dysregulated NOX activation increases superoxide production, accelerates the oxidative inactivation of NO, and may promote endothelial nitric oxide synthase (eNOS) uncoupling. In parallel, activation of nuclear factor kappa B (NF-κB) induces the expression of cytokines, chemokines, and adhesion molecules that promote leukocyte recruitment and endothelial inflammation. Diabetes-related endothelial dysfunction therefore reflects a self-reinforcing interplay among oxidative stress, impaired NO signaling, inflammation, and metabolic dysregulation rather than a simple deficiency of antioxidant capacity [1,2,3].

Blueberries, cranberries, strawberries, blackberries, and raspberries contain diverse anthocyanin glycosides of cyanidin, delphinidin, malvidin, pelargonidin, petunidin, or peonidin; their profiles vary substantially across species [4]. Blackcurrant and chokeberry are additional anthocyanin-rich sources examined in vascular research [5,6]. Some berry interventions improved brachial artery flow-mediated dilation (FMD), including a six-month blueberry trial in metabolic syndrome [7] and blueberry trials in healthy adults [8]. These findings do not imply consistent clinical efficacy: vascular responses were mixed, as detailed in Section 3.8. Aronia has also been studied for vascular effects in healthy men [5]. Responses vary with berry species, food matrix, composition, dose, duration, baseline vascular risk, medication use, and the method used to assess vascular function.

The biological effects of ingested anthocyanins cannot be explained solely by direct radical scavenging by the intact compounds. Following ingestion, anthocyanins undergo absorption, host conjugation, chemical degradation, and extensive transformation by the gut microbiota; the resulting microbial products may subsequently undergo phase II metabolism [9,10,11,12]. A human isotope-tracer study using ^13^C-labeled cyanidin-3-glucoside demonstrated extensive metabolism, with labeled products detected for up to 48 h after ingestion [12]. However, metabolites such as hippuric acid and several benzoic and phenylpropionic acids may also originate from other polyphenols, dietary substrates, or endogenous metabolic pathways [9,11,12]. Unless their origin is supported by appropriate analytical evidence, these compounds should be described as putative or berry-associated metabolites rather than as uniquely anthocyanin-derived.

Mechanistic studies have shown that selected blueberry-associated metabolites, tested at concentrations reported to circulate after blueberry consumption, provide greater protection against lipotoxicity-induced endothelial dysfunction than the parent anthocyanins examined [13]. Studies in diabetic endothelial cells and db/db mice further indicate that blueberry- or strawberry-associated metabolites and preparations can attenuate endothelial inflammation, leukocyte adhesion, NOX4-related oxidative stress, and vascular dysfunction [14,15,16]. More recent work implicates the gut microbiota in generating strawberry-derived metabolites associated with reduced vascular inflammation [17]. Nevertheless, whole berries contain numerous potentially bioactive constituents, and clinical findings obtained in healthy individuals or populations at cardiometabolic risk cannot be assumed to apply directly to patients with established diabetes.

Previous reviews have examined complementary aspects of anthocyanin bioactivity. Festa et al. [11] emphasized the vascular relevance of circulating phenolic metabolites, whereas Ahles et al. [18] evaluated randomized berry-anthocyanin interventions across vascular, cognitive and cardiometabolic outcomes. Mao et al. [19] examined glycemic and cardiovascular biomarkers in type 2 diabetes, although improvements in these measures do not establish improved endothelial function. More recent reviews have addressed the broader vascular mechanisms of anthocyanins [20] and their antidiabetic potential across biochemical pathways and human trials [21]. Building on these complementary perspectives, the present review focuses on diabetes-associated endothelial dysfunction, distinguishes diabetic evidence from findings in broader cardiometabolic risk populations, and critically examines metabolite provenance, physiological exposure and the strength of mechanistic inference.

This critical narrative review therefore evaluates preclinical and clinical evidence on the effects of berry anthocyanins and their confirmed or putative metabolites on endothelial dysfunction associated with diabetes and cardiometabolic risk. It separates direct evidence obtained in experimental models of diabetes and patients with established diabetes from broader translational evidence, examines the plausibility of metabolite-mediated mechanisms, and identifies priorities for future clinical research.

## 2. Literature Search and Narrative Synthesis

### 2.1. Search Strategy

This critical narrative review was informed by structured searches of MEDLINE via PubMed, Web of Science Core Collection, and Scopus. No publication-date restriction was applied, and the final searches were completed on 1 July 2026. Reference lists and forward citations of relevant publications were also examined selectively.

Search terms covered four concepts: berries; anthocyanins and their metabolites; diabetes and cardiometabolic risk; and endothelial outcomes. The representative search expression was: ((berry OR berries OR blueberry OR bilberry OR blackcurrant OR chokeberry OR aronia OR cranberry OR strawberry OR raspberry OR blackberry) AND (anthocyanin* OR anthocyanidin* OR phenolic metabolite* OR microbial metabolite*) AND (diabet* OR hyperglyc* OR insulin resistan* OR metabolic syndrome OR cardiometabolic OR lipotoxic*) AND (endothel* OR flow-mediated dilation OR vascular reactivity OR nitric oxide OR eNOS OR NADPH oxidase OR adhesion molecule*)).

The search was conducted in the title and abstract fields in PubMed and in the title, abstract, and keyword fields in Scopus. The topic field was used in Web of Science. Database-specific syntax was adapted without changing the underlying concepts, and no outcome-direction terms were applied. Study screening, selection and data extraction were performed by a single author. During study selection, the author rechecked the screening decisions. No independent verification by a second reviewer was performed.

The searches identified 298 records: 39 in PubMed, 144 in Scopus, and 115 in Web of Science. Records were deduplicated using DOI, PMID, and normalized-title matching. After removal of 45 duplicates, 253 unique records remained.

### 2.2. Eligibility and Study Selection

Eligible studies examined whole berries, anthocyanin-rich berry preparations, individual berry anthocyanins, or confirmed or putative metabolites. Human studies included adults with diabetes or elevated cardiometabolic risk. Preclinical evidence included diabetic or diet-induced metabolic animal models, ex vivo vascular preparations, and endothelial cells exposed to high glucose, palmitate, advanced glycation end products, inflammatory stimuli, or diabetic serum.

Priority was given to direct endothelial outcomes, including flow-mediated dilation, endothelium-dependent relaxation, NO production or eNOS signaling, endothelial ROS or NOX activity, adhesion and inflammatory markers, leukocyte–endothelial interactions, permeability, and glycocalyx integrity. Human reports with circulating endothelin-1 or adhesion-molecule measurements but no functional vascular endpoint were retained as indirect evidence and are identified as such in Section 3. For animal vascular preparations, phenylephrine-induced contraction and perivascular adipose markers alone were treated as contextual when acetylcholine served only to verify endothelial integrity and the intervention was not compared for an endothelial-specific outcome. Studies reporting only glycemic, lipid, blood pressure, systemic antioxidant, or nonspecific circulating inflammatory outcomes were treated as contextual evidence. Reviews and meta-analyses were used to identify primary studies and support interpretation but were not considered direct evidence. Protocols, editorials, registry entries without published results, and conference abstracts without sufficient data were excluded from the main synthesis.

**Figure 1:**
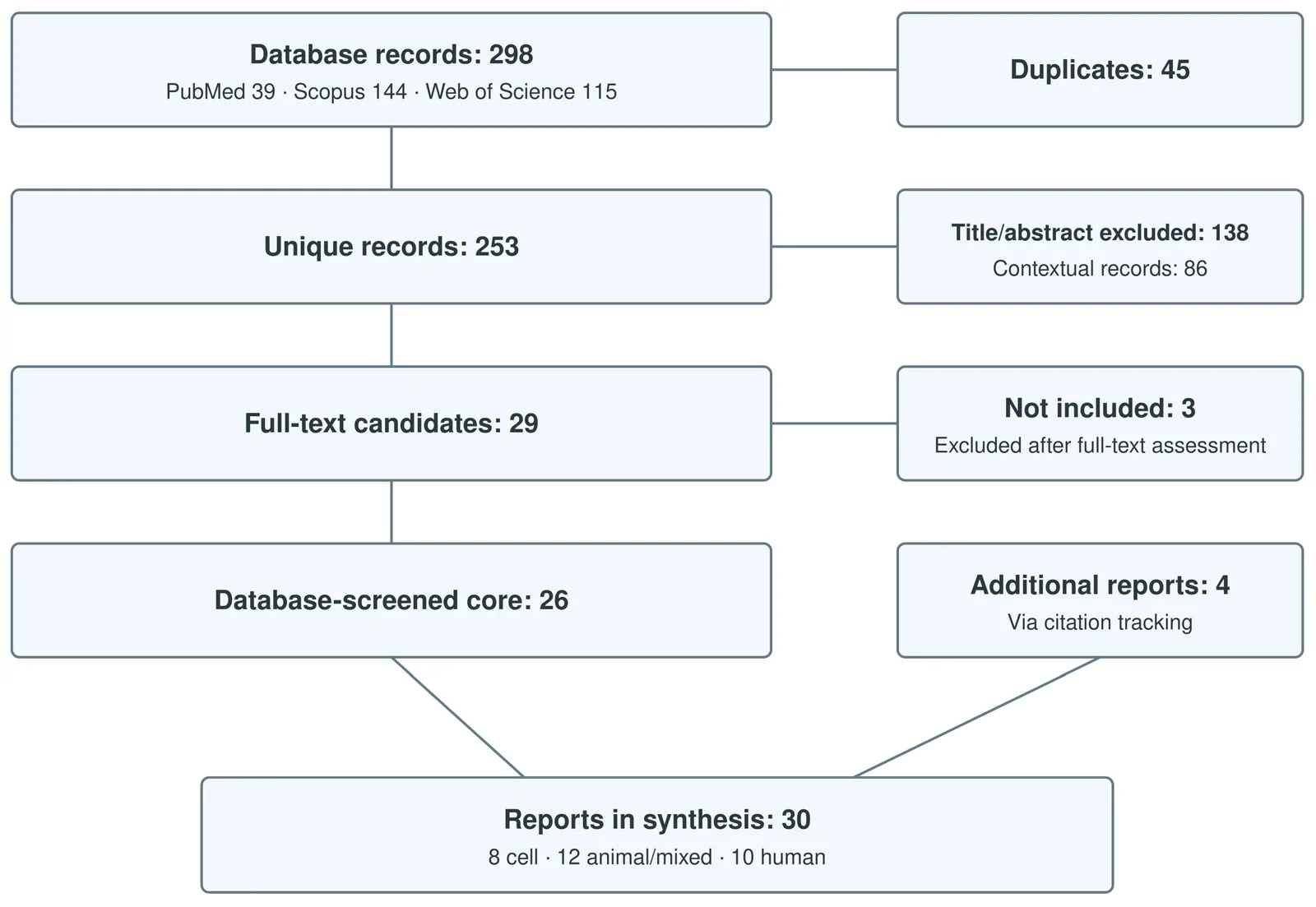
Study selection.

Following an initial screening of titles and abstracts from 253 unique records, 138 items were excluded and 86 were classified as contextual material; 29 were selected for full-text assessment. Twenty-six were included in the synthesis and three were excluded after full-text analysis. Four additional reports were identified through citation tracking and included in the synthesis; these reports were not among the 253 unique database records screened. In total, 30 reports were synthesized: eight cell-based, twelve animal or mixed, and ten human reports. The study selection process is illustrated in Figure 1. A record-level title and abstract screening sheet for 253 unique records, with title-and-abstract decisions and the record-level full-text decisions, is provided as Supplementary Table S1.

### 2.3. Data Extraction and Methodological Appraisal

Extracted information included study design, population or experimental model, berry intervention, anthocyanin composition and dose, exposure duration, comparator, animal sex and age, model induction, reported randomization and blinding, metabolite identity, analytical confirmation, and endothelial outcomes. For human studies, direct measures of endothelium-dependent dilation were distinguished from reactive-hyperemia measures of microvascular function and from indirect biomarkers or arterial-stiffness outcomes. Where reported, the outcome’s prespecified status, effect estimate, and confidence interval were noted. In vitro concentrations were compared with reported human circulating concentrations when pharmacokinetic data were available.

Interpretive limitations were recorded separately for human, animal, and cell-based studies without formal study-level risk-of-bias judgments or numerical quality scores. For trials, relevant domains included randomization, allocation concealment, blinding, missing vascular data, and prespecified outcomes; for animal studies, randomization, blinding, and sample-size rationale were considered. Cell studies were examined for preparation characterization, human-relevant concentrations, cell source and passage, controls, independent replication, and reporting completeness. These observations should not be interpreted as completed formal risk-of-bias assessments, including SYRCLE [22].

### 2.4. Narrative Synthesis

Evidence was classified as direct when it involved established diabetes, diabetic animals or endothelial cells, or a defined diabetes-related insult; translational when it involved cardiometabolic risk populations or metabolic models without established diabetes; and mechanistic-background evidence when it addressed metabolism or vascular biology without a diabetes-related context.

Studies were grouped by design, intervention, endothelial outcome, and proposed mechanism, with emphasis on the NOX–ROS, eNOS–NO, and NF-κB pathways and on the distinction between intact anthocyanins, confirmed or putative metabolites, and whole-berry effects. Following reporting principles for synthesis without meta-analysis [23], meta-analysis was not performed because of substantial heterogeneity in populations, interventions, experimental models, and endothelial outcomes.

## 3. Evidence Synthesis

### 3.1. Structure of the Evidence Base

The principal evidence formed three complementary streams: mechanistic studies using endothelial cells exposed to high glucose, palmitate, inflammatory stimuli, or a diabetic donor phenotype; animal studies using db/db or diet-induced metabolic models; and human interventions conducted mainly in metabolic syndrome or other cardiometabolic risk populations. No direct clinical evidence in patients with established diabetes and a direct endothelial endpoint was identified within the included studies.

### 3.2. Principal Studies Informing the Review

The principal synthesis comprised 30 reports: eight cell-based reports [13,14,24,25,26,27,28,29], twelve animal or mixed reports [6,15,16,17,30,31,32,33,34,35,36,37], and ten human reports [7,38,39,40,41,42,43,44,45,46]. The reports [7,39] concern related participants and represent one independent clinical investigation. The study by Bharat et al. [13] also included ex vivo artery experiments and is counted once. Four studies [15,17,36,38] were identified through citation tracking and added to the 26 reports included from database screening after revision.

### 3.3. Anthocyanin Sources and Exposure Characterization

Blueberry and strawberry interventions dominated the direct evidence. Blueberries contain complex mixtures of malvidin, delphinidin, petunidin, cyanidin, and peonidin glycosides, whereas strawberry anthocyanins are dominated by pelargonidin-3-glucoside. Aronia provides mainly cyanidin glycosides and has been studied extensively in vascular trials, although principally outside diabetic populations [5,40]. Variation in the berry matrix is mechanistically important because fiber, flavonols, proanthocyanidins, chlorogenic acids, ellagitannins, vitamins, and minerals may alter absorption, microbiota metabolism, or vascular responses.

Characterization was strongest when investigators quantified native compounds and circulating products with LC-MS/MS and authentic standards. In contrast, whole-fruit experiments cannot establish that an observed vascular effect was caused exclusively by anthocyanins. This limitation is particularly relevant to strawberries, in which ellagitannins and their urolithin metabolites may contribute to biological activity. This review accordingly identifies whole berries, polyphenol fractions, purified compounds, and administered metabolites separately in the evidence tables.

### 3.4. Bioavailability and Metabolite Formation

Intact anthocyanins exhibit rapid absorption and elimination, but total exposure is substantially underestimated when only parent glycosides are measured. The ^13^C-cyanidin-3-glucoside tracer study established that labelled degradation products and conjugates remain detectable across a prolonged sampling period [12]. Subsequent blueberry trials quantified a broad circulating metabolite profile and associated selected metabolites with acute and chronic FMD responses [8]. These observations support biological plausibility but do not by themselves prove that a correlated metabolite caused the vascular response.

Gut microorganisms convert anthocyanins and other berry polyphenols into smaller aromatic compounds, while enterocytes and liver generate glucuronidated, sulfated, and methylated derivatives [9,10,11,47]. In the strawberry HFD model, antibiotic treatment suppressed selected metabolite production and abolished the reduction in vascular inflammation, providing experimental support for microbiota-dependent mediation [17]. Even so, compounds such as hippuric acid remain nonspecific. Stable-isotope tracing of individual berry anthocyanins, combined with authentic analytical standards and microbiome perturbation, offers the strongest route to establish provenance.

**Figure 2:**
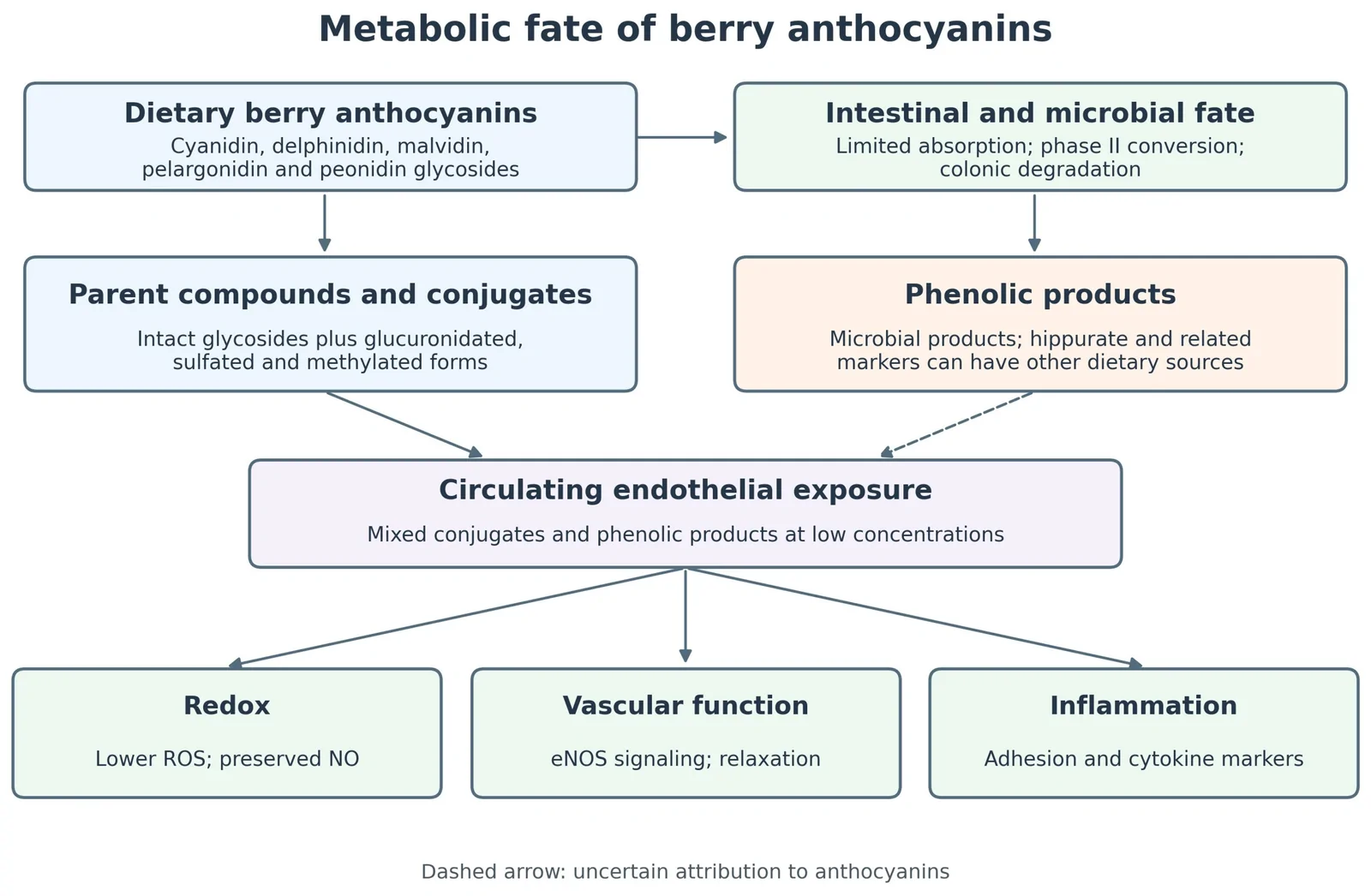
Metabolic fate of berry anthocyanins and possible endothelial responses. Dashed arrow denotes uncertain source attribution. eNOS—endothelial nitric oxide synthase; NO—nitric oxide; ROS—reactive oxygen species.

The metabolic fate of berry anthocyanins and the distinction between structurally attributable and nonspecific circulating phenolic products are summarized in Figure 2.

### 3.5. Endothelial Mechanisms of Action

Bharat et al. provided a central mechanistic observation: a mixture of blueberry-associated metabolites informed by human exposure data, but not the tested parent anthocyanins cyanidin-3-glucoside and malvidin-3-glucoside, preserved NO production under palmitate exposure [13]. Under these experimental conditions, this finding challenges the attribution of protection solely to the tested native anthocyanins. Complementary diabetic and cardiometabolic models linked berry exposure with lower oxidative and inflammatory signaling, improved endothelial repair, and better endothelium-dependent relaxation [6,16,24,30,31,32,33,34,35,36].

#### 3.5.1. Oxidative Stress and NADPH Oxidase Signaling

NOX-related redox changes were frequently reported in experimental models. In palmitate-treated human aortic endothelial cells, blueberry-associated metabolites reduced measured ROS and NOX4 mRNA expression [13]. Dietary blueberry was associated with lower vascular NOX4 mRNA expression in db/db mice [16], whereas strawberry supplementation altered NOX2 mRNA expression and inflammatory measures in a diet-induced metabolic model [17]. Cyanidin-3-glucoside also suppressed glycated-LDL-induced NOX4 protein upregulation and mitochondrial dysfunction in porcine aortic endothelial cells [24]. None of these expression measurements alone demonstrates direct inhibition of isoform-specific NOX activity.

These data are consistent with the established NOX–NOS imbalance in diabetes [2,3]. Reduced superoxide formation would be expected to preserve NO, limit peroxynitrite generation, and decrease redox-sensitive inflammatory signaling. However, most directly relevant experiments measured expression or bulk ROS rather than isoform-specific enzyme activity, eNOS coupling, tetrahydrobiopterin oxidation, or spatially resolved redox signaling. Claims that anthocyanins “restore redox homeostasis” should therefore remain conditional.

#### 3.5.2. Nitric Oxide, Inflammation and the Endothelial Surface

Across experimental models, studies reported attenuation of endothelial activation. Blueberry-associated metabolites reduced chemokine and adhesion-molecule expression and monocyte binding in lipotoxic human aortic endothelial cells [13]. In diabetic human aortic endothelial cells, related metabolites reduced inflammatory activation and increased cell-surface glycosaminoglycans, a potential glycocalyx-related measure [14]. Strawberry and blueberry interventions in diabetic mice reduced vascular inflammatory indices and leukocyte–endothelial interactions [15,16]. Whole-berry findings cannot isolate anthocyanins from other constituents.

These findings are compatible with redox-sensitive inflammatory signaling and reduced leukocyte recruitment, but they do not demonstrate that NF-κB activity is necessary for the observed response. Differences in stimuli, cell origin, metabolite mixtures, and analytical endpoints prevent quantitative pooling. Cell-surface glycosaminoglycans are a promising translational measure, but functional glycocalyx endpoints and berry-specific replication remain sparse.

### 3.6. Evidence from Endothelial Cell Models

Aboonabi et al. [29] examined a bilberry- and blackcurrant-derived anthocyanin preparation in primary human aortic endothelial cells from one donor with diabetes and one without diabetes. Treatment reduced H_2_O_2_-induced cytotoxicity, LPS-induced IL-6 release and caspase-1 activity, with a significant caspase-1 effect reported in diabetic cells, but it did not improve the GSH/GSSG ratio. These findings support cellular protection and anti-inflammatory activity; NF-κB activation and ROS production were not directly measured. The reported exposure of 50 µL/mL cannot be converted reliably to an anthocyanin mass or molar concentration because the stock concentration was not specified. Donor differences and the absence of functional vascular or circulating-metabolite measurements limit physiological interpretation.

Cell models provided an opportunity to compare parent anthocyanins with circulating metabolites. Bharat et al. exposed human aortic endothelial cells to palmitate and tested malvidin-3-glucoside, cyanidin-3-glucoside, and five phenolic metabolites at concentrations informed by human pharmacokinetic data after blueberry intake; equivalence to circulating exposure was not established for every mixture constituent [13]. The metabolite mixture, but not the parent glycosides under the tested conditions, reduced measured intracellular ROS, NOX4 mRNA expression, inflammatory gene expression, and monocyte adhesion while preserving NO production. This does not establish direct inhibition of NOX4 enzyme activity or prove NF-κB-dependent mediation. Its main limitations were the short exposure, use of a simplified metabolite mixture, and inability to reproduce repeated dietary exposure, tissue deconjugation, or interactions with plasma proteins.

Cutler et al. extended this metabolite-centered approach to primary aortic endothelial cells obtained from donors with diabetes [14]. Blueberry-associated phenolic metabolites decreased inflammatory activation and increased cell-surface glycosaminoglycans. The endothelial glycocalyx regulates permeability, leukocyte adhesion, mechanotransduction, and access of circulating proteins to the endothelial membrane. Restoration of glycosaminoglycans is therefore a plausible bridge between biochemical changes and vascular function. Nevertheless, the study did not establish which individual metabolite was necessary or sufficient, and donor-level variability and the clinical characteristics of the source material require consideration.

Kim et al. [37] provide complementary evidence for endothelial barrier protection in streptozotocin-induced diabetic rats. Oral *Vaccinium myrtillus* extract (100 mg/kg/day for 6 weeks; 36% anthocyanins, corresponding to 36 mg/kg/day) reduced retinal vascular leakage and VEGF expression and partly preserved ZO-1, occludin and claudin-5 without significantly changing blood glucose. This functional permeability endpoint supports protection of the blood–retinal barrier, but the complex extract and absence of circulating metabolite measurements preclude attribution to individual anthocyanins or metabolites.

In porcine aortic endothelial cells, Xie et al. showed that 30 µM cyanidin-3-glucoside attenuated glycated-LDL-induced NOX4 protein upregulation, superoxide generation, mitochondrial complex I/III dysfunction, and loss of cell viability [24]. The diabetes-related insult and direct RAGE–NOX–mitochondrial endpoints strengthen mechanistic relevance, although the concentration exceeded typical circulating concentrations of intact cyanidin-3-glucoside and the model used porcine cells.

High-glucose models supported overlapping antioxidant and vasoregulatory pathways. Blueberry anthocyanin extract, malvidin, malvidin-3-glucoside, and malvidin-3-galactoside reduced ROS and NOX4 protein expression while increasing SOD, HO-1, eNOS-related signaling, and NO in human umbilical vein endothelial cells [25]. In retinal endothelial models, related blueberry preparations reduced oxidative and inflammatory activation and, in a more recent study, modulated angiogenesis through the miR-33/GLCCI1 axis [26,28]. These experiments support protection of macrovascular and microvascular endothelial phenotypes, but concentration, stability, and spontaneous degradation were not always characterized sufficiently to assign activity to the nominal parent compounds.

The choice of metabolic insult materially affected interpretation. High-glucose models reproduce selected features of glucotoxic stress but require osmotic controls and sufficiently prolonged exposure. Palmitate models lipotoxicity but can cause nonspecific cytotoxicity depending on the fatty-acid-to-albumin ratio. Kowalska et al. [27] tested lingonberry fractions in separate hypertrophic adipocyte and TNF-α-stimulated HUVEC experiments; the endothelial assay did not use adipocyte-conditioned medium. These parallel assays do not establish adipocyte-to-endothelium mediation. Table 1 summarizes cell-based studies examining endothelial responses to berry preparations, anthocyanins, and berry-associated metabolites under diabetes-related metabolic stress.

### 3.7. Evidence from Diabetic and Cardiometabolic Animal Models

The twelve included animal or mixed studies linked molecular changes with vascular function under diabetic or cardiometabolic stress [6,15,16,17,30,31,32,33,34,35,36,37]. In alloxan-diabetic rats, a hydroalcoholic maqui extract improved acetylcholine-dependent aortic relaxation and NO bioavailability, although the extract contained multiple phenolic constituents and rutin was tested as a separate comparator [30]. In streptozotocin-diabetic apoE-deficient mice, dietary cyanidin-3-O-glucoside improved aortic relaxation, endothelial progenitor-cell function, endothelial repair, and atherosclerotic lesion burden [31].

Two Saskatoon berry studies in db/db mice addressed complementary mechanisms. Berry powder reduced monocyte adhesion to the aortic wall together with lower NOX4 protein expression and changes in inflammatory, adhesion, stress, and fibrinolytic markers [32]. A subsequent mixed mouse–cell study showed attenuation of endoplasmic-reticulum stress and unfolded-protein-response markers by Saskatoon berry powder in cardiovascular tissue and by cyanidin glycosides in glycated-LDL-treated endothelial cells [34]. These findings broaden the mechanistic framework beyond oxidative stress, but the whole-berry intervention prevents anthocyanin-specific attribution.

In obese Zucker rats, wild blueberry altered aortic contractile and relaxation responses and modulated NOS- and COX-related pathways [33]. Interpretation is not straightforward because the model displayed reduced phenylephrine contraction and exaggerated acetylcholine relaxation at baseline; the intervention partly normalized these atypical responses rather than simply increasing vasodilation.

Dietary blueberry in db/db mice improved mesenteric artery relaxation, lowered monocyte binding and vascular inflammatory markers, and reduced NOX4 mRNA expression [16]. Blackcurrant extract increased vascular eNOS staining and reduced histopathologic indices of vascular injury in diabetic KK-Ay mice [6]. These studies support direct diabetic vascular effects, although both used chemically complex preparations and incompletely reported several bias-control procedures.

Li et al. combined a high-fat-diet mouse model of type 2 diabetes with palmitate-exposed HUVECs and reported improved aortic relaxation and endothelial NO signaling after malvidin-3-O-glucoside, alongside lower arginase 1 expression [35]. Reversal of the cellular effect by ARG1 overexpression strengthened mechanistic inference, although the administered dose limits dietary translation. Strawberry-associated metabolites and feeding improved vascular inflammatory or endothelial outcomes in db/db mice [15]. In a high-fat-diet model, microbiota depletion reduced selected strawberry-associated metabolites and abolished the observed vascular anti-inflammatory effect [17]. Antibiotics can alter host physiology in addition to metabolite formation; neither whole-fruit experiment isolates anthocyanins as the causal constituents. Furuuchi et al. [36] reported restored acetylcholine-dependent iliac-artery relaxation in mice with diet-induced obesity after boysenberry polyphenols, alongside evidence involving purified anthocyanins, NO and eNOS dimerization; its direct vascular endpoint supports inclusion in the main synthesis. Other animal studies remain contextual [48,49,50,51]. Park et al. [50] reported vascular endothelial and inflammatory markers; because the present comparison emphasizes functional vascular measurements, its status remains a boundary of the classification. Table 2 presents animal and ex vivo studies assessing vascular effects of these interventions in diabetic and cardiometabolic models.

Table 2 compares sex, age, model induction, dose, duration, and reported bias-control procedures. Most studies used male animals; the aged female KK-Ay study [6] and the study with unreported sex [36] limit comparisons by sex and age. Genetic diabetic models, alloxan/STZ-induced diabetes, and diet-induced obesity or metabolic stress represent different disease contexts. Exposure ranged from 21 days to 16 weeks and included dietary percentages, drinking-water concentrations, and gavage doses, which cannot be compared as equivalent anthocyanin exposures. Random allocation was reported in seven of the twelve studies; blinding was not explicitly reported in the examined full texts. These reporting gaps do not prove that the procedures were omitted, but they limit assessment of internal validity and prevent attributing between-study differences to preparation or mechanism alone.

### 3.8. Human Intervention Evidence

Ten human reports represented nine independent clinical investigations because the acute postprandial report [39] analyzed a substudy related to the six-month trial [7]. These investigations involved metabolic syndrome, cardiovascular risk, hypercholesterolemic, prehypertensive, overweight, or elevated-blood-pressure populations rather than patients with established diabetes [7,38,39,40,41,42,43,44,45,46]. FMD assessed endothelium-dependent dilation, whereas reactive hyperemia assessed microvascular reactivity; arterial stiffness, circulating progenitor cells, endothelin-1, and inflammatory markers were interpreted as indirect or supporting outcomes. Table 3 distinguishes outcome class, principal result, and key design limitation. Effect estimates and confidence intervals are specified where verified from the primary report rather than inferred from a direction of change.

In a nonrandomized before–after study, two months of aronia extract reduced endothelin-1, blood pressure, lipids, and erythrocyte oxidative stress markers in participants with metabolic syndrome [41]. The absence of a concurrent control group and the small sample substantially limit causal inference. In a six-week randomized trial in 44 adults with metabolic syndrome, blueberry smoothies improved the reactive hyperemia index relative to placebo, whereas blood pressure and insulin sensitivity did not differ between groups [38]. The reactive hyperemia index reflects peripheral microvascular reactivity and should not be equated with brachial FMD. In a crossover trial of men with cardiovascular risk factors, six weeks of a wild-blueberry drink reduced oxidative DNA damage but did not significantly improve reactive hyperemia or other endothelial markers [42]. This null vascular result is important for balancing the evidence base.

A 12-week randomized trial of black raspberry extract in metabolic syndrome reported a lower radial augmentation index, increased circulating CD34/CD133-positive endothelial progenitor cells, and reductions in selected inflammatory markers [43]. These outcomes are vascular surrogates; neither stiffness nor progenitor cell counts directly measure endothelium-dependent dilation.

In a six-month randomized trial involving 115 participants with metabolic syndrome, one cup-equivalent of blueberries per day improved FMD (within-group change +1.45 percentage points; 95% CI 0.83–2.10; placebo change +0.39 percentage points; difference in changes +1.06 percentage points; *p* = 0.003 for the intervention effect), whereas the half-cup-equivalent dose had no reported effect [7]. FMD was a secondary outcome; insulin resistance was the prespecified primary outcome and did not improve. A postprandial substudy of the same trial population found favorable responses in selected metabolic and lipid measures after an energy-dense meal but no effect on FMD, pulse-wave velocity, augmentation index, or blood pressure [39]. These reports should not be counted as independent clinical replications.

In adults with moderate hypercholesterolemia, four weeks of strawberry powder did not change fasting FMD, but an acute 50 g dose increased FMD at 1 h; phenolic metabolites were associated with this acute response [45]. The post-intake response should not be interpreted as evidence of sustained adaptation or improved fasting endothelial function. In 102 prehypertensive adults, aronia supplementation improved arterial-stiffness measures but did not improve FMD, blood pressure, or lipids; metabolite and microbiome associations do not prove microbial mediation [40]. Daily blueberry intake for 12 weeks improved brachial FMD normalized to shear rate in postmenopausal women with above-normal blood pressure and was accompanied by lower oxidative stress [46].

In the AppleCOR trial, only the aronia infusion arm was relevant to the present review. It lowered P-selectin and improved the lipid profile, but the sustained improvement in ischemic reactive hyperemia was observed with the red-fleshed apple rather than the aronia infusion [44]. A separate postprandial strawberry trial in otherwise-healthy adults with obesity was retained as contextual evidence because the participants were not selected for a defined cardiometabolic disorder; it found no vascular benefit after a single high-fat meal [52]. Across the core human evidence, results ranged from null to favorable and cannot be extrapolated directly to established diabetes. Table 3 compares human interventions, distinguishing direct measures of endothelial function from other vascular outcomes and indirect markers.

Differences in exposure and outcome assessment may help explain the mixed clinical findings. Acute responses should be distinguished from sustained effects: strawberry intake improved FMD at 1 h but not fasting FMD after four weeks [45], whereas acute blueberry intake with an energy-dense meal did not improve FMD [39], despite a favorable six-month result [7]. Duration alone does not explain efficacy, since six-week blueberry trials yielded different reactive-hyperemia results [38,42]. Dose may contribute: 364 mg/day of blueberry anthocyanins improved FMD in the six-month trial, whereas 182 mg/day did not [7]. However, this within-study comparison does not establish a threshold across preparations; whole-berry powders, extracts and filtered infusions differ in composition, and extract mass is not equivalent to anthocyanin dose.

Participants also differed in baseline cardiometabolic status, including metabolic syndrome [7,38,39,41,43], cardiovascular risk [42], hypercholesterolemia [44,45], prehypertension [40] and postmenopausal status with elevated blood pressure [46]. These differences may modify responsiveness, but their contribution cannot be separated from differences in intervention and study design. FMD, reactive hyperemia, arterial stiffness and circulating biomarkers assess distinct aspects of vascular health, so a favorable result for one cannot compensate for a null result for another. Baseline impairment, endpoint selection and measurement timing should therefore be considered when comparing trials. These are plausible explanations for heterogeneity rather than demonstrated effect modifiers, and the findings remain applicable primarily to the studied cardiometabolic risk populations rather than established diabetes.

### 3.9. Metabolite Identity, Specificity and Plausible Exposure

Anthocyanin metabolites differ in source specificity. Intact anthocyanidin conjugates retain the flavonoid skeleton, whereas smaller phenolic acids and hippurate derivatives may arise from several dietary and endogenous substrates. Stable-isotope tracing can establish anthocyanin origin [12,53], but detection after berry intake alone cannot. Phenolic sulfates are distinct from conjugates of intact anthocyanidins.

Bharat et al. [13] and Cutler et al. [14] tested a defined five-metabolite mixture informed by human exposure data, partly obtained after sulfatase hydrolysis. Table 4 summarizes individual compounds, possible origins, experimental concentrations and human circulating benchmarks. The mixture’s activity does not identify the contribution of each constituent.

Human blueberry studies [7,39] report hippuric acid concentrations in the same micromolar range as the experimental exposure, but comparable exposure has not been established for all constituents. Absolute concentrations, changes from baseline and tracer Cmax must be distinguished; positional isomers, conjugation, protein binding and exposure duration further limit comparisons. Strawberry plasma changes [45] and phenolic profiles following aronia or blueberry intake [40,44,46] provide additional context without establishing equivalent endothelial exposure. The high-dose C3G tracer study [53] also shows that some parent-anthocyanin treatments substantially exceed measured circulating levels, although it does not represent an ordinary berry serving or exposure in diabetes.

In serum-transfer studies [15,16], metabolites were not quantified in the serum applied to endothelial cells, precluding compound-specific exposure estimates. Plasma measurements after enzymatic deconjugation [17] support microbial involvement but do not resolve intact conjugates or establish exclusive anthocyanin origin. These limitations should be considered when interpreting physiological relevance.

### 3.10. Consistency and Methodological Appraisal

Experimental reports suggest endothelial responses, whereas clinical efficacy in established diabetes remains untested within the included interventions. Cell studies often reported lower oxidative or inflammatory activation, and animal studies added functional vascular measurements. Several reports originated from related groups. The ten human reports represented nine independent investigations; Refs. [7,39] analyzed related participants with chronic and acute outcomes, respectively. Direct FMD findings were mixed. The RHI improvement in [38] provides a distinct microvascular signal, while the FMD benefit in [7] concerned a secondary rather than primary endpoint.

Appraisal was qualitative and reporting-based, not a completed RoB 2 or SYRCLE domain-by-domain assessment. Table 1, Table 2 and Table 3 identify limitations for individual reports: intervention specificity and exposure relevance in cell studies, model and functional endpoints in animal studies, and control group, sample size, outcome class, and relevant endpoint status in human studies. For animal studies, randomization, blinding, and sample-size rationale were not consistently reported; cell studies varied in osmotic controls, measured compound stability, and independent biological replication. Where a reporting item was unclear, no formal risk-of-bias judgment was inferred. These observations are interpretive limitations rather than standardized ratings.

Publication bias could not be assessed because studies were few and heterogeneous, and selective reporting is plausible when many biomarkers are measured. The direction and coherence of the evidence are therefore more informative than effect magnitude and do not establish clinical benefit. Table 5 summarizes the principal strengths and methodological limitations of each evidence stream.

### 3.11. Berry-Specific Evidence Profiles

#### 3.11.1. Blueberries and Bilberries

Blueberries provided the largest and most connected evidence stream, spanning whole fruit, extracts, parent anthocyanins, and circulating phenolic products. Their glycosides of malvidin, delphinidin, petunidin, cyanidin, and peonidin vary by cultivar, processing, and storage. This breadth supports triangulation but prevents direct dose equivalence among wild and cultivated fruit, powders, and purified extracts.

Blueberry-associated metabolites were tested in lipotoxic endothelial cells, diabetic donor cells, and db/db mice [13,14,16]. These models support pathway plausibility but do not equate the tested metabolite mixture or purified high-dose compounds with ordinary dietary exposure [24,25,26,28,35].

Human blueberry findings were mixed: RHI improved in one metabolic-syndrome trial [38]; secondary FMD improved in a separate six-month trial [7], whereas a related acute substudy found no FMD effect [39]. FMD also improved in postmenopausal women with elevated blood pressure [46]. These populations do not establish efficacy in diabetes; separate studies of monocyte oxidative stress and cardiometabolic biomarkers provide context [54,55].

#### 3.11.2. Strawberries

Strawberries are characterized by pelargonidin-3-glucoside and substantial ellagitannin content. Strawberry feeding improved endothelial function and vascular inflammation in db/db mice [15], but circulating-product experiments could not attribute activity to anthocyanins alone; urolithins and other ellagitannin-derived compounds may contribute. “Strawberry-associated metabolites” is therefore the more accurate term.

A strawberry trial in moderate hypercholesterolemia reported improved FMD at 1 h after intake but no improvement in fasting FMD after four weeks [45]. The acute response indicates a transient vascular effect at the measured time point and does not demonstrate sustained endothelial adaptation. Both anthocyanin and ellagitannin metabolite exposures merit measurement in future studies.

#### 3.11.3. Aronia and Blackcurrant

Aronia berries contain high concentrations of cyanidin glycosides together with procyanidins and chlorogenic acids. In metabolic syndrome, aronia extract lowered endothelin-1 and several cardiometabolic or oxidative stress measures in an uncontrolled study [41]. Controlled trials reported changes in arterial function and integrated these endpoints with metabolite or microbiome measurements [5,40]. In the AppleCOR trial, the aronia infusion lowered P-selectin and improved lipids, but the sustained reactive-hyperemia benefit was confined to the red-fleshed apple arm [44]. Standardized black chokeberry extracts also protected vascular reactivity in mouse aortic rings [51], although co-occurring constituents prevent exclusive attribution to anthocyanins. A 2026 crossover study assessed cerebral vascular function after aronia extract in older adults with overweight or obesity [56]; this endpoint is relevant to vascular health but is not equivalent to peripheral endothelial function.

Blackcurrants are characterized by delphinidin- and cyanidin-rutinosides and glucosides. The diabetic KK-Ay mouse study indicated vasculoprotective effects of blackcurrant extract [6]. Translation requires clear reporting of extract standardization and circulating metabolites, because rutinosides may differ from glucosides in absorption and microbial conversion. No sufficiently replicated set of diabetes-specific human trials reporting direct endothelial outcomes was identified.

#### 3.11.4. Raspberries, Blackberries, Lingonberries and Other Berries

Raspberries and blackberries contain cyanidin derivatives and variable amounts of ellagitannins. Black raspberry extract reduced augmentation index and increased circulating endothelial progenitor cells in metabolic syndrome [43]. The included boysenberry study reported improved endothelium-dependent vascular function in obese mice [36]. A mixed-berry intervention provided complementary evidence for vascular effects in a diet-induced metabolic model [49]; both interventions involved chemically complex polyphenol exposures. Maqui extract improved endothelium-dependent relaxation in alloxan-diabetic rats [30], whereas lingonberry fractions affected hypertrophic adipocytes and TNF-α-stimulated endothelial cells in separate assays [27]. These findings broaden the evidence beyond Vaccinium species but also reinforce the need to distinguish whole extracts from analytically defined anthocyanins.

Cranberry, elderberry, and haskap publications retrieved by the search largely emphasized composition, systemic antioxidant measures, glycemic enzymes, or outcomes outside direct endothelial function. These publications illustrate chemical diversity but contributed little direct evidence under the eligibility criteria. The absence of qualifying studies under these criteria does not establish an absence of biological activity.

### 3.12. Relationship Between Metabolic and Endothelial Outcomes

Glycemia, lipids, and blood pressure can influence endothelial stress, but they are not direct measures of endothelial function. Their relationship with NO signaling and tissue perfusion is bidirectional, so simultaneous improvement does not establish causal order.

Reviews have reported possible glycemic and lipid benefits in type 2 diabetes and substantial heterogeneity in vascular responses across berry trials [18,19]. This supports prioritizing endothelial and vascular outcomes in the principal synthesis rather than inferring vascular efficacy from systemic biomarkers.

Circulating inflammatory or adhesion markers likewise do not demonstrate endothelial target engagement. Stronger inference requires their combination with FMD, validated microvascular measures, ex vivo endothelial phenotyping, or glycocalyx-related outcomes.

## 4. Discussion

### 4.1. Integrated Interpretation of the Evidence

**Figure 3:**
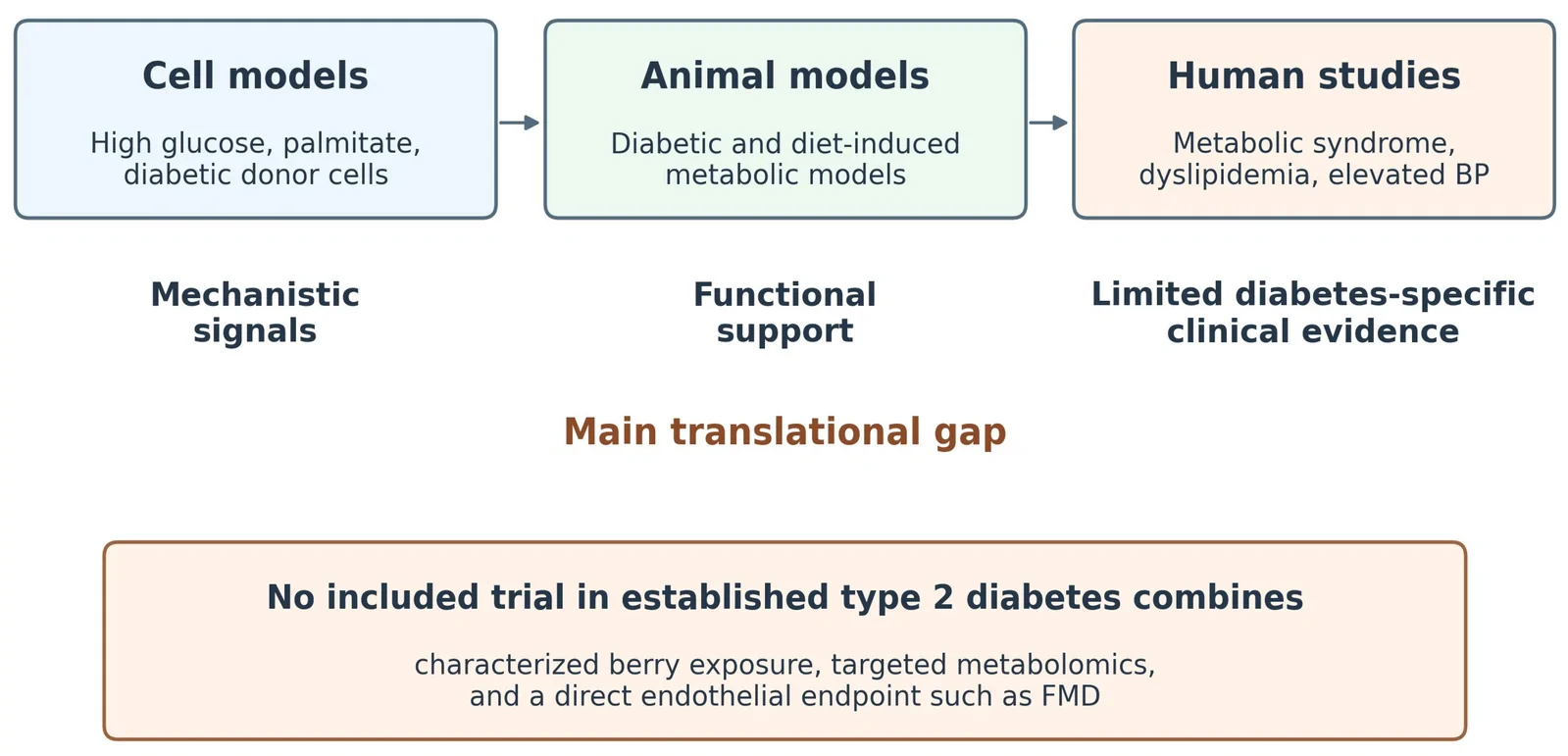
Evidence across experimental and human studies. No intervention in the included clinical set combined established type 2 diabetes, a chemically characterized berry preparation, targeted metabolomics, and a direct endothelial endpoint such as flow-mediated dilation (FMD). BP—blood pressure; FMD—flow-mediated dilation.

The main contribution of this synthesis is to distinguish what different study designs can establish. Across 30 reports, cell experiments offered control over compound identity and concentration, animal studies combined exposure with vascular testing, and nine independent human investigations addressed dietary relevance. Whole-berry interventions were more representative of dietary exposure but less informative about the active constituent; defined-metabolite experiments strengthened chemical attribution but simplified circulating exposure. Clinical comparisons in Section 3.8 show that favorable molecular or metabolic findings do not consistently translate into FMD or microvascular improvement and that dose, exposure duration, participant status, and outcome choice must be considered together. The contrast between mixed clinical responses and more consistent experimental signals identifies the central translational gap: mechanistic plausibility has not yet established vascular efficacy in patients with diabetes (Figure 3).

### 4.2. Proposed Mechanistic Framework

The mechanistic summary distinguishes molecular observations, functional outcomes, and causal validation. Lower ROS, changes in NOX or eNOS expression, and reduced inflammatory markers support molecular associations; they do not establish enzyme inhibition or pathway necessity. Preserved NO production, improved vasorelaxation, and reduced retinal barrier permeability support functional effects in the tested models, while human vascular findings remain mixed. Causal attribution requires targeted perturbation linked to the functional outcome, such as loss-of-function or rescue. Selected studies provide model-specific mechanistic evidence, including ARG1-related rescue [35], but this cannot be generalized to other pathways or berry preparations. Direct inhibition of isoform-specific NOX activity and the necessity of NF-κB signaling remain unestablished in most included models; preserved surface glycosaminoglycans alone do not demonstrate improved shear sensing.

Nrf2 activation, mitochondrial protection, and AGE–RAGE inhibition remain candidate pathways because they were not measured consistently. Associations between metabolites and FMD likewise require confirmation through physiologic dosing, dose–response analysis, and pathway perturbation.

**Figure 4:**
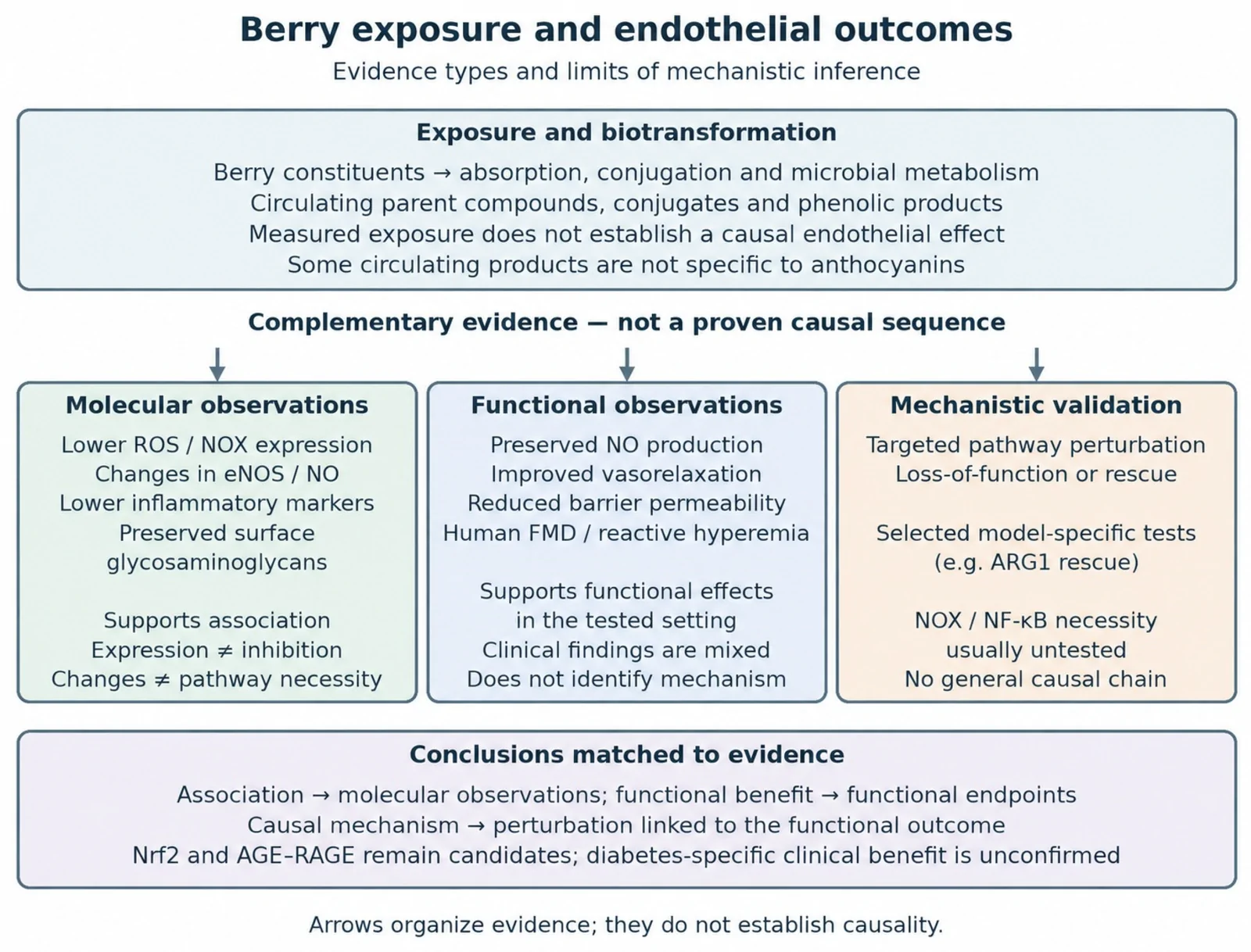
Evidence types and limits of mechanistic inference for berry-associated endothelial effects. Molecular changes support associations, functional endpoints support effects within the tested setting, and causal attribution requires targeted perturbation linked to a functional outcome. Selected model-specific validation does not establish a general causal chain. Arrows organize evidence and do not indicate proven causality. Diabetes-specific human vascular efficacy and clinical event reduction remain unconfirmed. ARG1—arginase 1, FMD—flow-mediated dilation, eNOS—endothelial nitric oxide synthase, NO—nitric oxide, NOX—NADPH oxidase, NF-κB—nuclear factor kappa B, Nrf2—nuclear factor erythroid 2-related factor 2, AGE–RAGE—advanced glycation end product–receptor for advanced glycation end products, ROS—reactive oxygen species.

Figure 4 separates exposure evidence from molecular observations, functional findings, and mechanistic validation and states the conclusion supported by each evidence type. Its arrows organize the evidence rather than imply a validated causal sequence from circulating metabolites to vascular benefit.

### 4.3. Parent Anthocyanins Compared with Their Metabolites

Parent glycosides may act in the gastrointestinal tract or during brief systemic exposure, whereas phase II conjugates and microbial products can circulate for longer. Local deconjugation may further modify exposure. Experiments should therefore test authentic circulating forms at pharmacokinetically justified concentrations and report conjugation status and analytical standards.

Terminology must remain conservative. Detection after berry consumption is not sufficient to establish exclusive anthocyanin origin. Hippuric acid, for example, reflects microbial and host metabolism of numerous aromatic precursors. The phrase “berry-associated phenolic metabolite” is more accurate unless stable-isotope or other source-specific evidence is available.

### 4.4. Clinical and Translational Implications

The comparisons in Section 3.8 support interpreting vascular responses within the studied population, preparation, exposure period, and endpoint. Findings in cardiometabolic risk populations do not establish efficacy in long-standing diabetes, where medication, renal function, glycemic control, and structural vascular damage may modify responsiveness.

Whole-berry, extract, and defined-metabolite studies therefore provide complementary evidence rather than interchangeable estimates of efficacy. Current evidence supports considering berries as a potential component of dietary risk reduction; diabetes-specific efficacy and anthocyanin-specific mechanisms require the studies outlined in Section 6.

## 5. Strengths and Limitations of the Review

This review separates direct diabetes-related evidence from broader cardiometabolic risk evidence and compares whole-berry, anthocyanin, and defined-metabolite exposures. Its principal strengths are the distinction between molecular associations, functional outcomes, and mechanistic validation and the comparison of experimental concentrations with human exposure benchmarks. Study selection decisions are documented in Supplementary Table S1.

The narrative design, single-author screening and extraction, and absence of formal certainty grading limit reproducibility and confidence in the synthesis. Heterogeneous populations, preparations, doses, and endpoints precluded meaningful meta-analysis. Small samples, related study populations, incomplete exposure characterization, and possible selective outcome reporting further limit effect-size inference and independent replication. These constraints require cautious interpretation of the comparisons and prevent generalization to established diabetes.

## 6. Future Research Directions

The highest priority is an adequately powered randomized trial in adults with established type 2 diabetes. A dietarily achievable, chemically characterized intervention should be compared with a matched control during stable standard therapy. FMD and a validated microvascular measure should be prespecified, and serial targeted metabolomics should support exposure–response analysis.

Mechanistic studies should use authentic standards, verify concentration and stability, and reproduce human metabolite ratios. Time-course and pathway-perturbation experiments should test whether NOX inhibition precedes restored NO signaling and reduced inflammatory transcription; glycocalyx outcomes warrant particular attention.

Stable-isotope-labeled anthocyanins combined with metagenomics and targeted metabolomics should identify the microbial enzymes, organisms, and circulating products derived from the administered compound. Causal studies should then test authentic metabolites within the human exposure range.

A future systematic update should register a protocol, archive complete strategies, avoid access restrictions, use duplicate screening and extraction, and report full-text exclusions. Shared standards for berry composition, metabolite annotation, endothelial outcomes, and physiologically relevant in vitro concentrations would improve future quantitative synthesis. Table 6 presents research priorities aimed at clarifying clinical efficacy, metabolite origin, and causal vascular mechanisms.

## 7. Conclusions

Across 30 reports, berry anthocyanins, berry preparations, and berry-associated metabolites were associated with endothelial-protective effects in experimental settings relevant to diabetes and cardiometabolic risk; ten human reports represented nine independent investigations, with vascular findings ranging from null to favorable. The most frequent experimental associations involved lower measured ROS or NOX expression, preserved NO signaling, reduced inflammatory or adhesion markers, and improved vascular relaxation. The necessity of NOX and NF-κB pathways remains incompletely tested. Circulating metabolites may contribute more directly than selected parent anthocyanins, but whole-berry studies cannot attribute causality exclusively to anthocyanins, and several phenolic metabolites lack source specificity. Direct clinical evidence in established diabetes remains insufficient and requires well-controlled, metabolite-informed trials with prespecified endothelial outcomes.

Short-term tolerability findings are reassuring for some preparations: no adverse events were reported in a 12-week black-raspberry extract trial [43], while aronia extract was generally well tolerated over 12 weeks, with no serious adverse events but two events considered possibly related to treatment [40]. These findings do not establish long-term safety or the safety of higher-dose concentrated extracts, particularly in established diabetes. In practice, the evidence supports including whole berries as part of a healthy dietary pattern rather than replacing them with anthocyanin supplements. It does not establish that supplements are superior to whole berries, that a specific preparation or dose treats diabetic endothelial dysfunction, or that berry intake prevents vascular complications or clinical cardiovascular events. Such preparations should not replace established treatment.

## Figures and Tables

**Table 1:** Core cell-based studies relevant to diabetes-associated endothelial dysfunction.

Study	Model and Metabolic Insult	Berry Exposure	Endothelial Outcomes	Interpretive Limitation
Xie et al. [24]	Porcine aortic endothelial cells; glycated LDL	C3G 30 µM; 30 min pretreatment; endpoints at 2–60 h; redox dose response 0–50 µM	ROS, NOX4 protein expression, RAGE, mitochondrial complexes and viability	High parent-compound concentration; porcine cells
Bharat et al. [13]	Human aortic endothelial cells; palmitate	C3G 0.012 µM; M3G 0.016 µM; five-metabolite mixture: VAS 0.075, IVAS 0.075, BAS 0.7, HHA 3 and HA 5 µM; cells 6 h; aortic segments 16 h	ROS, NOX4 mRNA expression, NO, inflammatory genes and monocyte adhesion	Short exposure; simplified, nonspecific phenolic mixture; NOX4 mRNA, not enzyme activity; ex vivo arteries also tested (counted once)
Cutler et al. [14]	Primary diabetic human aortic endothelial cells	Five-metabolite mixture: VAS 0.075, IVAS 0.075, BAS 0.7, HHA 3 and HA 5 µM; 3 days	Inflammation and surface glycosaminoglycans	Mixture effects; limited donor characterization; surface glycosaminoglycans are not a functional glycocalyx test
Huang et al. [25]	Human umbilical vein endothelial cells; high glucose	BAE, Mv, Mv-3-glc, Mv-3-gal: each 5 µg/mL for 24 h before 30 mM glucose for 24 h	ROS, NOX4 protein expression, SOD, HO-1, eNOS and NO	Nominal parent-compound stability uncertain
Huang et al. [26]	Human retinal capillary endothelial cells; high glucose	BAE, Mv, Mv-3-glc, Mv-3-gal: each 10 µg/mL for 24 h before 30 mM glucose for 24 or 48 h	ROS, antioxidant enzymes, NOX4 protein expression and inflammation	Microvascular model; dose translation uncertain
Kowalska et al. [27]	HUVECs; TNF-α 10 ng/mL; separate hypertrophic adipocyte experiments	ACN or PP: 0.1, 1 or 10 µg/mL for 3 h before TNF-α for another 3 h	Endothelial ROS and inflammatory dysfunction	Direct TNF-α model; separate adipocyte assays; fraction doses are not individual-compound molar concentrations
Luo et al. [28]	Human retinal endothelial cells; 30 mM glucose	BAE 10 µg/mL (10 mg/L) for 24 h before 30 mM glucose for 48 h	ROS, MDA, SOD, tube formation and miR-33/GLCCI1 signaling	Extract mass concentration; constituent-specific doses and human exposure equivalence not established
Aboonabi et al. [29]	Primary human aortic endothelial cells from one diabetic and one non-diabetic donor; H_2_O_2_ (75 µM) or LPS (1 µg/ml)	Medox anthocyanin preparation from bilberry and blackcurrant; 50 µL/ml (stock concentration not specified); 80 mg anthocyanins/capsule; GSH/GSSG assessed at 6 and 24 h; LPS stimulation for 1 h before treatment	Lower H_2_O_2_-induced cytotoxicity and LPS-induced IL-6 release; reduced caspase-1 activity (significant in diabetic cells); no improvement in GSH/GSSG	One donor per group; volumetric dose not convertible to µg/ml or µM; NF-κB and ROS not directly measured; no functional vascular endpoint or metabolite exposure data

C3G—cyanidin-3-O-glucoside, M3G—malvidin-3-O-glucoside, VAS—vanillic acid-4-sulfate, IVAS—isovanillic acid-3-sulfate, BAS—benzoic acid-4-sulfate, HHA—hydroxyhippuric acid, HA—hippuric acid, BAE—blueberry anthocyanin extract, Mv—malvidin, Mv-3-glc—malvidin-3-glucoside, Mv-3-gal—malvidin-3-galactoside, ACN—lingonberry anthocyanin fraction, PP—lingonberry non-anthocyanin polyphenol fraction, HUVECs—human umbilical vein endothelial cells, LDL—low-density lipoprotein, ROS—reactive oxygen species, NOX4—NADPH oxidase 4, NADPH—reduced nicotinamide adenine dinucleotide phosphate, RAGE—receptor for advanced glycation end products, SOD—superoxide dismutase, HO-1—heme oxygenase 1, eNOS—endothelial nitric oxide synthase, NO—nitric oxide, TNF-α—tumor necrosis factor alpha, MDA—malondialdehyde, miR-33—microRNA-33, GLCCI1—glucocorticoid-induced transcript 1, H_2_O_2_—hydrogen peroxide, LPS—lipopolysaccharide, IL-6—interleukin-6, GSH—reduced glutathione, GSSG—oxidized glutathione, NF-κB—nuclear factor kappa B, mRNA—messenger RNA.

**Table 2:** Included animal and ex vivo studies linking berry exposure with endothelial outcomes under diabetic or cardiometabolic stress. Findings marked as expression or association do not establish enzyme activity or pathway necessity.

Study	Model	Intervention	Main Vascular Findings	Evidence Classification
Petersen et al. [15]	Male db/db mice; 7 weeks at dietary intervention (6 weeks at purchase); genetic leptin-receptor deficiency; ex vivo vascular and endothelial assessment	2.35% freeze-dried strawberry diet, 10 weeks; serum transfer: 5% for 3 days	Lower vascular inflammation and improved endothelial outcomes	Direct diabetic model; serum metabolites not measured; serum-transfer effects are not compound-specific; random allocation not reported; blinding not reported
Petersen et al. [17]	Male C57BL/6J mice; 7 weeks at intervention (6 weeks at purchase); high-fat diet (60% kcal fat), with/without antibiotics; diet-induced metabolic stress	2.35% freeze-dried strawberry diet, 12 weeks; with/without antibiotics	Lower vascular inflammation; benefit lost with antibiotic treatment	Plasma phenolics measured after deconjugation; main figures show relative levels; antibiotic effects are not compound-specific proof; random allocation not reported; blinding not reported
Fuentes et al. [30]	Male Wistar rats; 8–11 months, as reported; alloxan-induced diabetes (80 mg/kg); aortic rings	Maqui hydroalcoholic extract: 50 mg/kg per gavage, twice daily for 21 days; separate rutin comparator: 30 mg/kg twice daily	Improved endothelium-dependent relaxation and NO bioavailability	Direct diabetic; chemically complex extract; random allocation reported; blinding not reported
Zhang et al. [31]	Male apoE-deficient mice; 8 weeks at induction; STZ 55 mg/kg/day for 5 days; diabetic atherosclerosis model	C3G: 0.2% (*w*/*w*) in AIN-93 diet for 6 weeks	Improved aortic relaxation, EPC function and endothelial repair	Direct diabetic; purified anthocyanin; random allocation reported; blinding not reported
Zhao et al. [32]	Male db/db mice; 7 weeks at intervention (6 weeks at purchase); genetic leptin-receptor deficiency	Saskatoon powder: 5% (*w*/*w*) for 4-week vascular analyses; dose ranging from 0.2–20% for up to 5 weeks	Lower monocyte adhesion, NOX4 protein expression and vascular inflammatory markers	Direct diabetic; whole-berry exposure; random allocation reported; blinding not reported
Vendrame et al. [33]	Male obese Zucker rats (fa/fa); 8 weeks at intervention; genetic obesity, not chemically induced diabetes; studied at 16 weeks	8% (*w*/*w*) wild-blueberry powder in diet for 8 weeks; powder contained 1.5% (*w*/*w*) anthocyanins	Partial normalization of aortic contraction/relaxation and NOS/COX pathways	Cardiometabolic model; atypical baseline reactivity; random allocation reported; blinding not reported
Zhao et al. [34]	Male db/db mice; 7 weeks at intervention (6 weeks at purchase); genetic leptin-receptor deficiency; glycated-LDL-treated endothelial cells	5% (*w*/*w*) Saskatoon powder for 4 weeks; HUVECs: C3G 30 µM or C3Ga 100 µM, 30 min pretreatment; gLDL endpoints at 4–16 h	Lower cardiovascular and cellular ER stress/UPR markers	Mixed diabetic in vivo/in vitro evidence; high parent-compound concentrations; whole-berry effects cannot be assigned to C3G alone; random allocation reported; blinding not reported
Horie et al. [6]	Female KK-Ay mice; 3 years old, as reported; genetic diabetic model with high-fat feeding	1% or 3% blackcurrant extract powder in high-fat diet for 9 weeks	Higher vascular eNOS and less vascular remodeling/injury	Direct diabetic; histologic endpoints; random allocation not reported; blinding not reported
Petersen et al. [16]	Male db/db mice; 7 weeks at intervention (6 weeks at purchase); genetic leptin-receptor deficiency	3.8% freeze-dried blueberry diet, 10 weeks; serum transfer: 5% for 3 days	Lower NOX4 mRNA expression and monocyte binding; improved mesenteric relaxation	Direct diabetic model; serum metabolites not measured; whole-fruit and serum effects do not establish anthocyanin-specific causality; random allocation reported; blinding not reported
Li et al. [35]	Male C57BL/6J mice; 6–8 weeks at purchase plus 1-week acclimation; high-fat diet (60% kcal fat) for 16 weeks; diabetes defined by fasting glucose >11.1 mmol/L; palmitate-treated HUVECs	M3G: 50 or 150 mg/kg by oral gavage every other day for 16 weeks; HUVECs: 20, 50 or 100 µM for 24 h after 24 h palmitate exposure	Improved aortic relaxation and NO signaling through ARG1 inhibition	Direct diabetic; purified compound and ARG1 rescue; high administered dose limits dietary translation; random allocation reported; blinding not reported
Furuuchi et al. [36]	C57BL/6NCr mice; sex not reported; high-fat diet from 4 weeks; intervention from 12 weeks; assessed at 18–20 weeks; diet-induced obesity, not established diabetes	Boysenberry polyphenols: 0.1% in drinking water from age 12 to 18–20 weeks; HUVECs: polyphenols or anthocyanin fraction 10 µg/mL (NO: 6 h; ROS: 2 h pretreatment then 15-min palmitate)	Improved acetylcholine-dependent relaxation; higher NO and eNOS dimerization; lower ROS	Cardiometabolic model; fraction mass dose is not an individual-compound concentration; functional vascular endpoints; random allocation not reported; blinding not reported
Kim et al. [37]	Male Brown Norway rats; 6 weeks at purchase plus 1-week acclimation; STZ 60 mg/kg once; treatment after confirmation of diabetes; retinal barrier model	*Vaccinium myrtillus* extract: 100 mg/kg/day by oral gavage for 42 days; 36% anthocyanins (36 mg/kg/day)	Lower retinal vascular leakage and VEGF expression; partial preservation of ZO-1, occludin and claudin-5; no significant glycemic improvement	Direct diabetic model; functional blood–retinal barrier permeability endpoint; complex extract; circulating metabolites not measured; random allocation not reported; blinding not reported

C3G—cyanidin-3-O-glucoside, C3Ga—cyanidin-3-O-galactoside, M3G—malvidin-3-O-glucosid, STZ—streptozotocin, apoE—apolipoprotein E, LDL—low-density lipoprotein, gLDL—glycated LDL, HUVECs—human umbilical vein endothelial cells, EPC—endothelial progenitor cell, NO—nitric oxide, ROS—reactive oxygen species, eNOS—endothelial nitric oxide synthase, NOS—nitric oxide synthase, NOX4—NADPH oxidase 4, NADPH—reduced nicotinamide adenine dinucleotide phosphate, COX—cyclooxygenase, ER—endoplasmic reticulum, UPR—unfolded protein response, ARG1—arginase 1, VEGF—vascular endothelial growth factor, ZO-1—zonula occludens-1, mRNA—messenger RNA, AIN-93—American Institute of Nutrition 1993 rodent diet, *w*/*w*—weight/weight. db/db, fa/fa, KK-Ay, C57BL/6J and C57BL/6NCr denote rodent genotypes or strains.

**Table 3:** Core human studies assessing endothelial or vascular outcomes.

Study	Population and Design	Intervention	Vascular or Surrogate Outcome	Endpoint Class and Study-Level Limitation
Broncel et al. [41]	25 adults with metabolic syndrome; uncontrolled before–after study	Aronia extract 100 mg three times daily (300 mg/day) for 2 months; anthocyanin proportions reported, absolute anthocyanin dose not established	Lower endothelin-1, blood pressure and oxidative stress markers	Indirect endothelin-1 marker; uncontrolled before–after design (*n* = 25); no diabetes-specific inference
Stull et al. [38]	44 adults with metabolic syndrome; 6-week double-blind RCT	45 g/day freeze-dried blueberry powder in two smoothies; 580.6 mg anthocyanins/day (290.3 mg/smoothie); 6 weeks	Reactive hyperemia index improved versus placebo (adjusted change +0.32 ± 0.13 vs. −0.33 ± 0.14; *p* = 0.0023); no blood-pressure or insulin-sensitivity difference	Peripheral microvascular RHI, secondary outcome; 44 randomized, RHI analyzed in 40 (22 blueberry, 18 placebo); no brachial FMD
Riso et al. [42]	18 men with cardiovascular risk; 6-week crossover trial	25 g/day freeze-dried wild-blueberry drink; 375 mg anthocyanins/day; 6 weeks	No significant improvement in reactive hyperemia or endothelial markers	Microvascular reactive hyperemia and indirect markers; small crossover study (*n* = 18); no significant vascular improvement
Jeong et al. [43]	51 adults with metabolic syndrome; 12-week RCT	Unripe black-raspberry extract 750 mg/day (4 × 187.5-mg capsules) for 12 weeks	Lower augmentation index and increased circulating EPCs	Arterial stiffness and progenitor-cell surrogates; no direct dilation endpoint; *n* = 51
Curtis et al. [7]	115 adults with metabolic syndrome; 6-month double-blind RCT	75 or 150 g/day blueberry equivalent; 182 or 364 mg anthocyanins/day; 6 months	FMD: within-group change +1.45 percentage points (95% CI 0.83–2.10), versus +0.39 with placebo; difference in changes +1.06 percentage points (intervention *p* = 0.003) at 150 g/day; no effect at 75 g/day	Direct FMD, secondary outcome; primary insulin-resistance outcome unchanged; 115 randomized, FMD analyzed in 96; related substudy [39]
Huang et al. [45]	34 adults with moderate hypercholesterolemia; crossover RCT	50 g/day freeze-dried strawberry; 141.7 mg anthocyanins/day; 4-week crossover periods; acute testing at 1 h	No chronic FMD change; acute FMD improvement at 1 h	Secondary FMD outcome: acute increase at 1 h; no fasting change after four weeks; *n* = 34
Curtis et al. [39]	45 adults with metabolic syndrome; postprandial RCT	26 g freeze-dried blueberry with meal; 150 g fresh equivalent; 364 mg anthocyanins; assessment over 24 h	No effect on FMD, pulse-wave velocity, augmentation index or blood pressure; selected metabolic responses improved	Direct FMD null after single exposure; postprandial substudy (*n* = 45) related to [7], not independent replication
Le Sayec et al. [40]	102 prehypertensive adults; 12-week RCT	One 500-mg aronia capsule/day for 12 weeks; 105.9 mg individually quantified polyphenols; 4.65% anthocyanins (calculated 23.25 mg/capsule)	Improved arterial stiffness; no significant change in FMD	FMD measured with no significant change; improved stiffness is a distinct outcome; *n* = 102
Woolf et al. [46]	32 postmenopausal women with elevated blood pressure; 12-week RCT	22 g/day freeze-dried blueberry; 224 mg anthocyanins/day; 12 weeks	Improved shear-rate-normalized brachial FMD; lower oxidative stress	Direct endothelial function measurement; small sample (*n* = 32), postmenopausal women without diagnosed diabetes
Pedret et al. [44]	121 hypercholesterolemic adults; aronia arm *n* = 40; 6-week RCT	1 L/day filtered cold infusion prepared from 50 g aronia powder for 6 weeks; anthocyanin-matched to red-fleshed apple comparator	Lower P-selectin and improved lipids; no sustained reactive-hyperemia benefit attributable to aronia	Aronia arm (*n* = 40): P-selectin and lipid changes; no sustained microvascular reactive-hyperemia benefit

RCT—randomized controlled trial, RHI—reactive hyperemia index, FMD—flow-mediated dilation, EPCs—endothelial progenitor cells, CI—confidence interval.

**Table 4:** Representative compounds: possible origins, experimental concentrations and human exposure benchmarks.

Compound	Possible Origin	Experimental Exposure	Human Circulating Benchmark	Interpretive Limitation
Vanillic acid-4-sulfate	Host sulfation of vanillic acid; several phenolic precursors	0.075 µM in mixture [13,14]	Fasting plasma Δ +0.00100 µM after 4-week strawberry intake [45]; grouped serum methoxybenzoic acid sulfates Δ +0.0104 µM [7]	Plasma Δ is not an absolute exposure; between-treatment *p* = 0.09 [45]. Grouped serum isomers are unresolved [7].
Isovanillic acid-3-sulfate	Host sulfation of isovanillic acid; several phenolic precursors	0.075 µM in mixture [13,14]	Fasting plasma Δ +0.00086 µM after 4-week strawberry intake [45]; grouped serum methoxybenzoic acid sulfates Δ +0.0104 µM [7]	Plasma Δ is not an absolute exposure; grouped isomers [7] cannot establish a constituent-specific match.
Benzoic acid-4-sulfate	Host sulfation of a hydroxybenzoic acid product; source not unique	0.7 µM in mixture [13,14]	No directly comparable value verified in the human reports examined [7,39]	Human hippuric acid-sulfate is a different compound and cannot serve as its benchmark.
Hydroxyhippuric acid	Host glycine conjugation of hydroxybenzoic products; microbial metabolism may contribute	3 µM in mixture [13,14]	4-Hydroxyhippuric acid: 0.01616 µM at 6 h; 0.01411 µM at 24 h [39]	Experimental name does not specify isomer; these values cannot establish a direct exposure match.
Hippuric acid	Microbial aromatic metabolism and host glycine conjugation; multiple precursors	5 µM in mixture [13,14]	2.721 µM at 6 h; 3.984 µM at 24 h [39]; six-month Δ +2.468 µM [7]	Same micromolar range; nominal medium and total serum concentrations are not equivalent exposures.
Cyanidin-3-glucoside; malvidin-3-glucoside	Intact parent anthocyanins	C3G 0.012 µM; M3G 0.016 µM [13]; C3G 30 µM [24,34]; M3G 20–100 µM [35]	C3G tracer Cmax: 0.141 µM [53]	Parent exposures vary markedly; high micromolar treatments [24,34,35] require caution in dietary translation. Tracer dose: 500 mg C3G.
Protocatechuic acid; phloroglucinaldehyde	B-ring and A-ring degradation products, respectively; source-specific when labeled	No isolated-compound treatment in the experiments summarized here	Labeled tracer Cmax: 0.146 and 0.582 µM, respectively [53]	Provenance of labeled products does not establish origin of all unlabeled molecules.

C3G—cyanidin-3-O-glucoside, M3G—malvidin-3-O-glucoside, Cmax—maximum measured circulating concentration, Δ—change from baseline. All concentrations are expressed in µM. Human values represent absolute postprandial concentrations [39], changes from baseline [7,45], or mean serum Cmax following a 500 mg labeled C3G dose [53]; these measures are not directly interchangeable. “No directly comparable value verified” indicates that no matching value was confirmed in the sources examined.

**Table 5:** Reporting-based methodological appraisal of the evidence base; entries are not formal study-level risk-of-bias judgments.

Evidence Stream	Principal Strengths	Recurring Limitations	Overall Interpretation
Human clinical studies	Randomization; direct FMD in several trials; achievable food doses	No included intervention in established diabetes; heterogeneous and partly secondary endpoints; related clinical reports [7,39]	Mixed results; possible benefit in selected vascular outcomes
Animal studies	Integrated metabolism, microbiota and vascular function	Randomization/blinding and sample-size rationale often incompletely reported; whole-fruit causality unclear	Coherent functional support
Endothelial cell studies	Pathway testing and comparison of compounds/metabolites	Dose and chemical stability concerns; short exposure; expression often measured instead of pathway activity	Strong mechanistic plausibility
Metabolomics	Links exposure with circulating chemical signatures	Annotation uncertainty; correlations do not establish mediation	Useful for prioritizing causal tests

FMD—flow-mediated dilation.

**Table 6:** Priority design features for the next generation of studies.

Research Need	Recommended Design	Essential Measurements	Main Question Resolved
Clinical efficacy in diabetes	Multicenter double-blind RCT in established type 2 diabetes	FMD, microvascular function, HbA1c, medication and adverse events	Does a characterized berry intervention improve endothelial function?
Chemical attribution	Stable-isotope anthocyanin study	Serial plasma and urine targeted LC-MS/MS	Which circulating products truly derive from anthocyanins?
Causal vascular mechanism	Human-relevant metabolite mixtures with pathway perturbation	NOX activity, eNOS coupling, NO, NF-κB, adhesion and glycocalyx	Which pathways are necessary for protection?
Microbial mediation	Gnotobiotic or depletion–rescue design	Metagenomics, enzyme activity and labeled metabolites	Which microbial functions generate active products?
Response heterogeneity	Prespecified stratified clinical analysis	Diet, sex, renal function, drugs, microbiome and metabotype	Who responds and why?
Evidence synthesis	Prospectively registered update with duplicate screening	Open strategies, extraction and risk-of-bias decisions	Can comparable effects be pooled reliably?

RCT—randomized controlled trial; FMD—flow-mediated dilation; HbA1c—glycated hemoglobin; LC-MS/MS—liquid chromatography–tandem mass spectrometry; NOX—NADPH oxidase; NADPH—reduced nicotinamide adenine dinucleotide phosphate; eNOS—endothelial nitric oxide synthase; NO—nitric oxide; NF-κB—nuclear factor kappa B.

## Data Availability

A record-level title and abstract screening sheet for 253 unique records, with title-and-abstract decisions and the record-level full-text decisions, is provided as Supplementary Table S1.

## Supplementary Materials

The following supporting information can be downloaded at: https://www.mdpi.com/article/10.3390/molecules31203618/s1, Table S1: Study selection and screening decisions.
